# Pathological classification of Fuchs endothelial corneal dystrophy into several types and their relationships with CTG18.1 expansion repeats

**DOI:** 10.1002/path.70044

**Published:** 2026-02-25

**Authors:** Hanielle Vaitinadapoulé, Daria Onitiu, Corantin Maurin, Gauthier Travers, Emmanuel Crouzet, Oliver Dorado‐Cortez, Sylvain Poinard, Zhiguo He, Fabien Forest, Edouard Ollier, Renaud Touraine, Philippe Gain, Jean‐Marc Perone, Gilles Thuret, the French Fuchs Study Group (FFSG), Diane Bernheim, Christophe Chiquet, Vincent Borderie, Tristan Bourcier, Jean‐Louis Bourges, Frédéric Chiambaretta, Béatrice Cochener, Louis Arnould, Florian Baudin, Catherine Creuzot, Vincent Daien, Alexandre Denoyer, Bernard Duchesne, Nicolas Duquesne, Pierre Fournie, Anne‐Sophie Gauthier, Philippe Gain, Gilles Thuret, Louis Hoffart, François Majo, Marc Muraine, Romain Mouchel, Jean Marc Perone, Jean Claude Quintyn, Alexandra Rabot, Alain Saad, Damien Gatinel, Pierre‐Yves Santiago, Jean‐Michel Bosc, David Touboul, Bertrand Vabres

**Affiliations:** ^1^ Laboratory ‘Biology, Engineering and Imaging for Ophthalmology’, BiiO, Faculty of Medicine, Health Innovation Campus University Jean Monnet Saint‐Priest en Jarez France; ^2^ Genetics Department University Hospital Saint‐Etienne France; ^3^ Ophthalmology Department University Hospital Saint‐Etienne France; ^4^ Pathology Department University Hospital Saint‐Etienne France; ^5^ Clinical Research Unit University Hospital Saint‐Etienne France; ^6^ Ophthalmology Department Centre Hospitalier Regional Metz‐Thionville France

**Keywords:** corneal endothelium, Descemet's membrane, Fuchs endothelial corneal dystrophy, extracellular matrix, phenotypes, classification, CTG18.1 trinucleotide repeat expansion

## Abstract

Late‐onset Fuchs endothelial corneal dystrophy (FECD) is the most common primary disease of the corneal endothelium and the leading indication for corneal transplantation in Western countries. It is characterized by progressive accumulation, over two to three decades, of extracellular matrix (ECM) components in Descemet's membrane (DM), leading to the formation of abnormal excrescences, known as guttae, and additional DM layers. Clinical forms and evolutionary profiles vary widely among patients. FECD is strongly associated with intronic CTG trinucleotide repeats (TNRs) in the transcription factor 4 (*TCF4*) gene. We analysed 500 DMs removed during keratoplasty for FECD across 25 European centres to identify different anatomopathological forms of the disease. Following flat mounting and dehydration, the samples were digitized using transmitted light microscopy and independently assessed by three independent readers. A total of ten parameters – six related to guttae and four on other forms of ECM – were scored. Principal component analysis and an unsupervised clustering method separated three clusters from these parameters. In addition, manual classification was performed by grouping samples with major common features. The number of TNRs in *TCF4* was analysed by short tandem repeat (STR)‐ and triplet repeat primed‐polymerase chain reaction (TP‐PCR) for 109 patients. We found that (1) five FECD phenotypes exist; (2) guttae and other ECM structures were radially arranged in 95% of samples; (3) 33% of samples exhibited peripheral radial striae that corresponded to a hypertrophied form of similar structures present in healthy corneas; and (4) patients with fewer than 50 TNRs had only two out of five phenotypes and had a significantly higher number of peripheral radial striae (94% versus 49%, *p* < 0.001). Taken together, these new findings demonstrate the existence of different FECD phenotypes; reveal that lesions affect both the centre and the periphery of the endothelium; and suggest that radial deposits may be produced by pathological cells migrating from the periphery towards the centre. © 2026 The Author(s). *The Journal of Pathology* published by John Wiley & Sons Ltd on behalf of The Pathological Society of Great Britain and Ireland.

## Introduction

Late‐onset Fuchs endothelial corneal dystrophy (FECD) is by far the most prevalent primary disease affecting the corneal endothelium, and represents the leading indication for corneal graft in Western countries [1]. Its most characteristic examination and histology feature is the presence of small lesions referred to as ‘guttae’ – abnormal extracellular matrix (ECM) deposits forming rounded excrescences in Descemet's membrane (DM). Guttae gradually accumulate over two to three decades, starting from the centre of the dome of the DM (or, in some cases, the paracentre), growing towards its periphery. In parallel, premature corneal endothelial cell (CEC) death occurs. When disease progression is unfavourable, the endothelium is unable to regulate stromal hydration, leading to permanent corneal oedema and, eventually, significant impairment of visual acuity. Severe visual impairment is treated with endothelial keratoplasty, which involves replacing the central part of the endothelium with healthy endothelium from a donor cornea. Clinical classifications of FECD severity rely on the estimated number of guttae, the diameter of the confluent plaque [2, 3, 4], and the presence or not of corneal suboedema or oedema [5].

The mechanisms involved in premature CEC death and abnormal ECM production are gradually being deciphered, and FECD is strongly associated with an intronic CTG trinucleotide repeat (TNR) expansion in the transcription factor 4 (*TCF4*) gene [6], despite significant variation in prevalence (ranging from 76–79% of patients in Caucasian patients [7] to 26% in Japanese patients [8]). Several aspects of this mutation remain poorly understood, including the involvement of TNR expansion in guttae development.

Recently, we described novel structures at the periphery of the DM in FECD, termed peripheral striae. These appear as corpuscle‐like structures with a radial organization resembling pathological long extensions of Hassall–Henle warts [9]. We further suggested that dysfunction in the centripetal migration of pathological cells could play a role in FECD pathophysiology [10]. These observations required confirmation in a larger cohort.

The highly variable expressivity of FECD, in terms of both severity and speed of progression, is widely recognized by clinicians. From a clinical standpoint, this broad spectrum justifies the need for accurate classification of each cornea in order to provide personalized care. In basic, translational, and clinical research, this heterogeneity represents an obstacle as it limits the development of specific strategies.

To date, the anatomopathological characteristics of FECD have been described and gradually refined over time using mainly corneal cross‐sections observed under optical microscopy [11] and transmission electron microscopy [11, 12, 13], including serial block face scanning electron microscopy [14]. Flat mounts analysed by immunofluorescence [15] or scanning electron microscopy have also added to our understanding [16], although they are less frequently used. To decipher the clinical heterogeneity of FECD, we present a novel approach to FECD pathology by systematically analysing 500 flat‐mounted DMs from FECD patients to identify the types and frequency of ECM lesions and their possible associations. We then propose a new histological classification based on their organizational patterns and compare the genetic characterization of TNR expansion among groups.

## Materials and methods

### Ethical approval

The handling of tissues adhered to the tenets of the Declaration of Helsinki (2024 revision) [17] ensuring donor confidentiality, and was approved by the Ethics Committee of the St‐Etienne University Hospital (IRB_IORG0007394, Ref_IRBN1142021/CHUSTE).

### Human tissue samples

Human tissue samples were collected from 25 hospitals by 29 surgeons (supplementary material, Table S1). In this study of surgical specimens, the only clinical data authorized for collection were age and sex. Our objective was to analyse DMs only, excluding CECs. The only inclusion criterion was a diagnosis of FECD at a stage requiring keratoplasty. Each clinician had the discretion to choose the diagnostic criteria from the following list: presence of guttae on slit lamp and specular microscopy; signs of endothelial insufficiency; absence of other aetiologies; personal and family history; long duration of evolution. We collected the central endothelium, approximately 8 mm in diameter, during the first step of the graft, called descemetorhexis (supplementary material, Figure S1). Samples were immediately immersed in either balanced salt solution (BSS; Alcon, Rueil‐Malmaison, France), or sterile water to rapidly destroy residual CECs, or 4% paraformaldehyde (PFA). Samples were stored at room temperature and sent to the laboratory for analysis. Only one specimen was considered for bilateral cases. Additionally, peripheral blood samples (8 ml) were collected from 109 patients and genetic analyses were performed at two centres (St‐Etienne and Metz).

### Sample preparation and imaging

Each DM was mounted flat on a glass slide (Series 2; Trajan Scientific and Medical, Ringwood, Australia) under an operating microscope with the guttae facing upward. The slides were quickly air‐dried and stored in an airtight box at −20 °C until observation using transmitted light with bright field illumination (IX81; Olympus, Tokyo, Japan) equipped with a ×4 objective and ×1.6 zoom, and a CMOS camera (ORCA‐Flash4.0 LT Digital; Hamamatsu Photonics, Hamamatsu City, Japan). The mosaic of images (pixel ratio 0.98461 pixels/μm) was stored in TIFF format.

From a collection of approximately 1,000 DMs, 545 DMs were selected exclusively on the quality of the sample (complete disc, if possible, in one piece). Of these, 500 DMs were used to establish classifications, and 45 additional DMs were used to analyse relationships between phenotype and genotype. The collection of 500 images is publicly available in the Zenodo repository (see Data availability statement).

### Classification by systematic analysis of elementary lesions and clustering

We described the characteristic elementary lesions and their distribution across the surface. Based on a training cohort of 60 DMs, we established ten simple criteria, achievable without image analysis tools, to describe all possible lesions: six for guttae and four for other components of the ECM [furrows and an embossed appearance of the DM, and structures in the posterior fibrillary layer (PFL)] (Table 1). All lesions were observed from the endothelial side. As no other histological layer is described at this level, we considered the PFL to be the entire extracellular matrix that attenuated the contours of the drops until they disappeared (buried guttae). These manual readings were performed by three independent readers [HV, GT, and FM (see Acknowledgements)] blinded to the clinical data (age and gender), sample origin, and genetic background. Discrepant scores were then reviewed in a consensus meeting to reach a final score. We verified that, between the three readers, we were able to distinguish between small‐ and large‐diameter guttae and between different areas (for example, more or less than half of the DM area occupied by guttae). We adopted new terminology to describe three elements rarely described until now in FECD: (1) ‘peripheral striae’, previously cited [10], were by definition always located at the edge of the sample and interrupted by the surgical tear; (2) ‘curly fibres’, defined as fibres in the PFL wrapped around the guttae at varying densities; and (3) ‘guttae bands’, defined as very long intertwined bands of guttae with no notable orientation. These three structures are illustrated in supplementary material, Figure S2.

**Table 1:** Classification system for each elementary lesion observable on Descemet's membrane of Fuchs endothelial corneal dystrophy specimens.

Criterion (abbreviation)	Grades				*n* (%)
Guttae number (Gnum)	0: free surface > surface covered by guttae				68 (13.6)
	1: free surface = surface covered by guttae				231 (46.2)
	2: free surface < surface covered by guttae				201 (40.2)
Guttae shape (Gsha)	0: only or mostly round				433 (86.6)
	1: balanced mixture of round and deformed guttae				61 (12.2)
	2: only or mostly deformed				6 (1.2)
Guttae diameter (Gdia)	0: only or mostly small				38 (7.6)
	1: balanced mix of small and large diameters				448 (89.6)
	2: only or mostly large				14 (2.8)
Guttae overall pattern (Gpat)		Outer area	Intermediate area	Centre	
	0 centripetal gradient	0/+	++	+++	91 (18.2)
	1 rich periphery	+++	0/+	++	26 (5.2)
	2 homogeneous	+/++/+++	+/++/+++	+/++/+++	33 (6.6)
	3 intermediate ring	+	+++	+/++	340 (68.0)
	4 unclassifiable				10 (2.0)
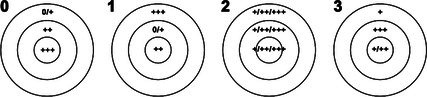				
Guttae radial orientation* (Grad)	0: absent				24 (4.8)
	1: present in one or two quadrants (= 90° or 180°)				134 (26.8)
	2: present in more than two quadrants (> 180°)				342 (68.4)
Guttae band (Gban)	0: absent				438 (87.6)
	1: present				62 (12.4)
‘Curly fibres’^†^ (Curl)	0: absent				100 (20.0)
	1: present but not the dominant structure				350 (70.0)
	2: abundant				50 (10)
Amorphous extracellular matrix (Amor)	0: absent				65 (13.0)
	1: present but not the dominant structure				409 (81.8)
	2: abundant				26 (5.2)
Radial orientation* of other structures (excluding guttae) (Orad)	0: absent				24 (4.8)
	1: present in one or two quadrants (= 90° or 180°)				112 (22.4)
	2: present in more than two quadrants (> 180°)				364 (72.8)
Peripheral striae^‡^ (Pstr)	0: absent				337 (67.4)
	1: present in one or two quadrants (= 90° or 180°)				112 (22.4)
	2: present in more than two quadrants (> 180°)				51 (10.2)

* Radial orientation defined as a significant alignment of at least ten elements in a straight line.

^†^ Initially described in the endothelial periphery of healthy subjects by Vrabec [18].

^‡^ Organization that we recently described [10].

Three parameters were quantified by image analysis (Fiji, https://imagej.net/software/fiji/downloads). The minimum and maximum Feret diameters of each DM were analysed to determine whether the frequency of peripheral striae located at the edge of the sample was underestimated in the smallest specimens. We also measured the diameter of the guttae in a random sample of DMs (seven in each of the three categories of guttae diameter distinguished by naked eye reading according to the criteria described in Table 1). We manually measured the maximum possible number of guttae within a 200‐μm‐wide band, extending from one edge of the sample to the other, passing through the centre, using the straight‐line selection tool in the Fiji software. Guttae with unclear contours (potentially due to the cell layer and/or cell debris in PFA‐fixed specimens) were excluded from analysis. We estimated that only 10–20% of the area of PFA‐fixed specimens was not accessible for reliable measurement.

### Manual classification

After noting significant similarities and differences between the DMs, we also carried out a manual classification, establishing two rules: (1) identify a single dominant characteristic as often as possible; if not possible, (2) select a maximum of two or three characteristics. This classification was performed independently by two observers (HV and GT). The groups and their intersections were represented using a proportional Venn diagram [19], generated in R (https://www.r-project.org).

### Genotyping

To investigate histological differences between TNR expansion‐positive and TNR expansion‐negative samples, we simultaneously characterized the genetic profiles and histological phenotypes for 109 patients selected at random. This cohort included 64 patients from the series of 500 DMs used to establish the classifications, and 45 additional patients classified using the established rules. Details on the two PCR techniques used are presented in supplementary materials and methods, Figure S3 and Tables S2–S4.

### Statistical analyses

Data are presented as mean ± SD, median (min–max). Data with a normal distribution were compared using parametric tests. Otherwise, a non‐parametric test was used. A *p* value less than 0.05 was considered the significance threshold unless otherwise stated. Principal component analysis (PCA) was performed on the ten manual classification variables, using an oblimin rotation, as it was assumed that residual correlations might exist between the principal components (PCs).

Two‐step clustering analysis, i.e. pre‐clustering followed by hierarchical clustering, was applied on the ten variables of the manual classification to automatically identify the optimal clustering number based on the silhouette width. Distances were calculated using log‐likelihood, and clustering was performed by Schwarz's Bayesian criterion [20]. Statistical analyses and graphs were performed using IBM SPSS Statistics software 30.0.0.0 (https://www.ibm.com/fr-fr/products/spss-statistics) except for the violin plots, which were generated using Statistics Kingdom (https://www.statskingdom.com).

## Results

### General characteristics and influence of pre‐analytical conditions

Each centre collected 21 ± 24 DMs (median 14, from 2 to 99). Patients had a mean age of 70 ± 9 years (median 71, range 41–95 years), with no significant age differences between centres (*p* = 0.331). Overall, 66.4% were women and 31.4% were men (2.2% missing data). Age between men and women did not differ significantly (*p* = 0.741). The minimum and maximum Feret diameters were 8.3 ± 1.1 and 9.5 ± 1.1 mm, respectively. The influence of the three transport liquids on the grading of elementary lesions is detailed in supplementary material, Figure S4 and Table S5, and can be summarized as follows: No morphological changes in the various ECM abnormalities were noted at the level of our observations. However, the DMs in PFA had lower scores for three of the ten items (amorphous ECM; guttae radially aligned; other structures radially aligned), suggesting that the fixed residual cells interfered with the observation of the finest structures (masking effect). For instance, radially aligned guttae were observed in more than two quadrants in 56.3% of PFA‐fixed specimens versus 78.9% in water and 66.7% in BSS (*p* < 0.001). Other radially aligned structures were found in more than two quadrants in 56.9% of PFA‐fixed specimens versus 80.3% in water and 81.7% in BSS (*p* < 0.001).

### Elementary lesions: descriptive data

The frequency distribution of individual lesions is detailed in Table 1 (right column) and can be summarized as follows: We identified a high number of guttae covering at least half of the Descemet surface in 86.4% of cases (grades 1 and 2 of item guttae number, GNum). These were predominantly round (86.6%) and presented a balanced combination of small and large guttae (grade 1 of item guttae diameter, Gdia) in 89.6% of cases. The majority (68.0%) of DMs showed a pattern with a central area of guttae surrounded by an even denser ring of guttae and then a much less dense area of guttae (grade 3 of item guttae pattern, Gpat).

**Figure 1:**
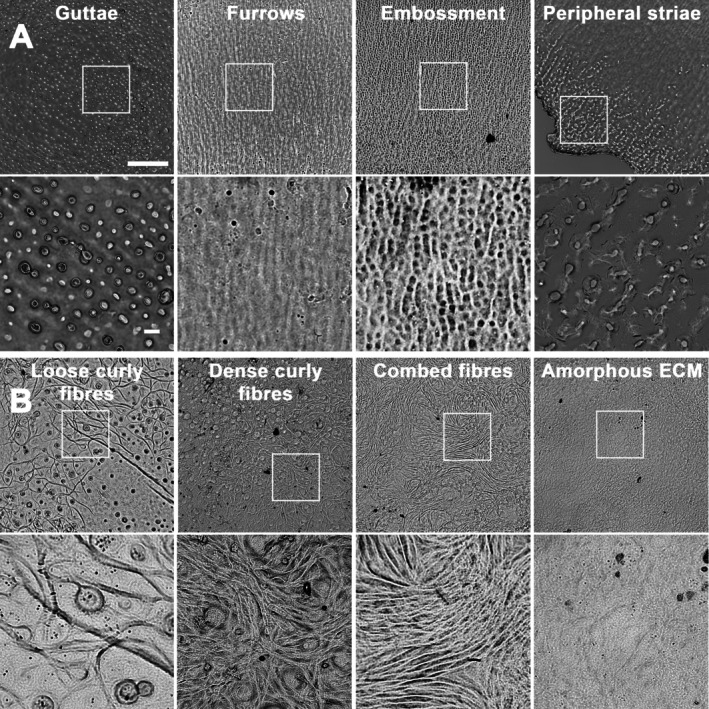
Structure of the guttae, Descemet's membrane furrows and embossing, peripheral striae, and the posterior fibrillar layer (PFL) at low and high magnifications (×10 and ×40 objectives). (A) The four radially organized structures. (B) Different structures composing the PFL. Scale bars: 200 μm for low‐magnification and 20 μm for high‐magnification images.

We identified three structures with a radial organization (Figure 1A): (1) In 95.2% of DMs, guttae (regardless of their diameter) were perfectly aligned, forming radially oriented lines that could be present over a variable surface area, limited to one or two quadrants in 26.8% of cases and extending to three or four quadrants in 68.4% of cases. These aligned guttae could co‐exist with randomly distributed ones. (2) Two structures (furrows or ‘embossment’ in the DM) also adopted a radial alignment in 95.2% of DMs with a variable extent over 360°. These were significantly associated with radial guttae (Spearman's coefficient = 0.255, *p* < 0.001). (3) Peripheral striae were present in 32.6% of cases. They were found around 360°, though they were sometimes visible in only one, two, three, or all four quadrants (possibly depending on the centration of the surgical dissection). Samples without peripheral striae were significantly smaller (minimum and maximum Feret diameters, 8.04 ± 1.01 and 9.24 ± 1.00 versus 8.86 ± 1.06 and 10.19 ± 0.97, *p* < 0.001 for both diameters) than those with striae, suggesting that we may have underestimated the percentage of striae on DMs that were small in diameter. There was no significant association between the radial organization of the guttae and the presence of peripheral striae (*χ* ^2^, *p* = 0.973).

We also identified two other ECM abnormalities (Figure 1B) that were located mainly (but not exclusively, as shown by the existence of a group of outliers) in the centre of the DM, above the guttae that often remained visible through transparency: (1) Curly fibres, with more or less tight loops, were present in 80.0% of cases and were the dominant structure (in terms of surface area) in 10.0%. They also appeared as combed fibres, although more rarely. (2) A layer of amorphous ECM covering the guttae was observed in 87.0% of cases and was the dominant structure in only 5.2% of cases. Only 5.8% (29/500) of DMs had neither of these two forms of abnormal ECM.

### Elementary lesions: distribution of the diameter of the guttae

**Figure 2:**
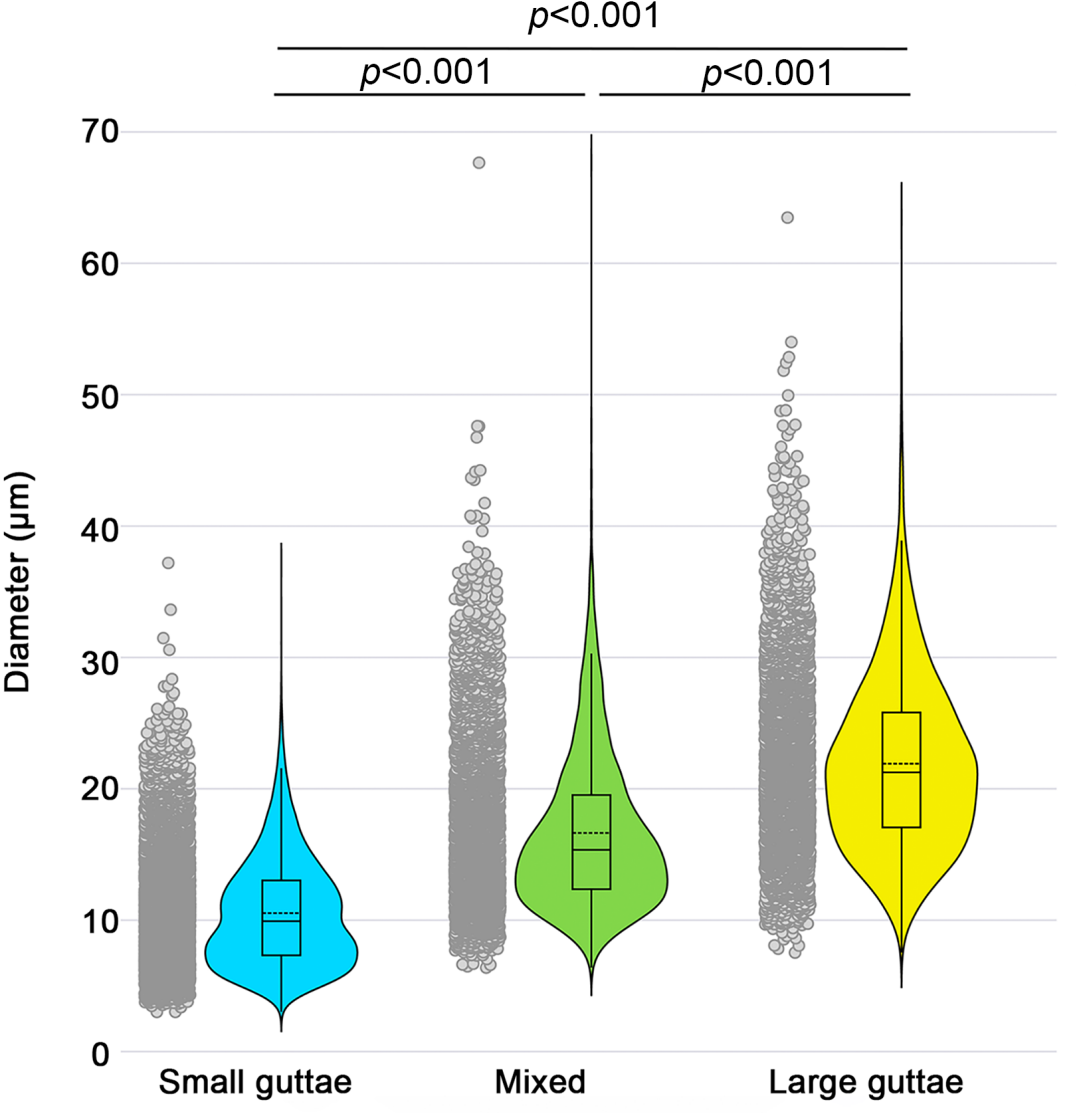
Guttae diameter. Scatter, violin, and box plots for the three categories of guttae diameter, separated according to our manual classification.

The diameters, measured on 15,841 guttae, were 10.5 ± 4.12 μm (median 9.93, range 3.0–37.2 μm), 16.6 ± 5.9 μm (median 15.4, range 6.4–67.6 μm), and 21.9 ± 6.6 (median 21.3, range 7.5–63.5 μm) for the small, mixed, and large guttae groups, respectively (*p* < 0.001). Two‐by‐two tests showed significant differences (Bonferroni correction) (Figure 2). This quantitative result indicated that our subjective classification of diameters effectively separated the three groups, and that the mixed group seemed to correspond to guttae of intermediate diameter rather than to two distinct populations. In all groups, the diameter range was very wide, confirming the diversity of intra‐ and inter‐patient diameters.

### Histological phenotypes by principal component analysis and clustering

**Figure 3:**
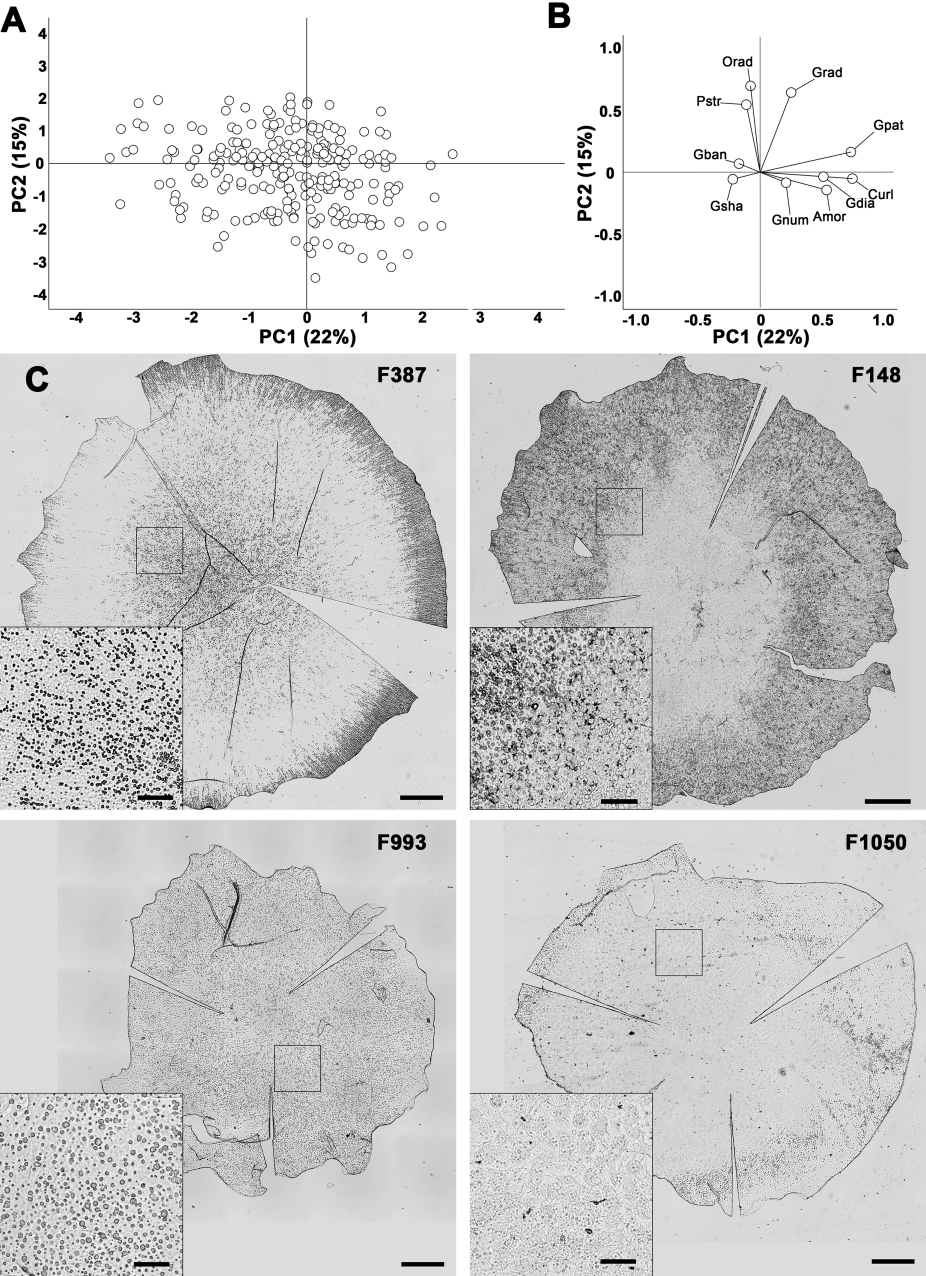
Graphical representation of the results of principal component analysis. (A) Projection of samples in the space constructed from the first two principal components (PCs). (B) Contribution of the ten variables to each of the first two PCs. Amor, amorphous ECM; Curl, curly ECM; Gban, bands of guttae; Gdia, guttae diameter; Gnum, guttae number; Gpat, global guttae pattern; Grad, radially aligned guttae; Gsha, guttae shape; Orad, other radially aligned structures; Pstr, peripheral radial striae. (C) Example of four Descemet membranes located at the extremes of the factorial planes of the first two PCs. Full‐section images are available in the online image collection (see the Materials and methods section for access information). F387 is notable for the presence of peripheral radial striae; F148 for the large elliptical centre of confluent guttae surrounded by aligned and radial guttae; F993 for the presence of only small, round guttae; and F1050 for the almost complete coverage by curly structures that mask the guttae. Scale bar, 1 mm. For each Descemet's membrane, a 1 mm^2^ inset shows characteristic lesions, highlighting diversity and effective separation using the principal components. Scale bar, 200 μm.

Four principal components (PCs) were obtained, explaining 59% of the total variance. PC1 corresponded broadly to the characteristics of the ECM, PC2 to the radial nature of the different structures, and PCs 3 and 4 to the characteristics of the guttae. PCs 1 and 2 are shown in Figure 3.

**Figure 4:**
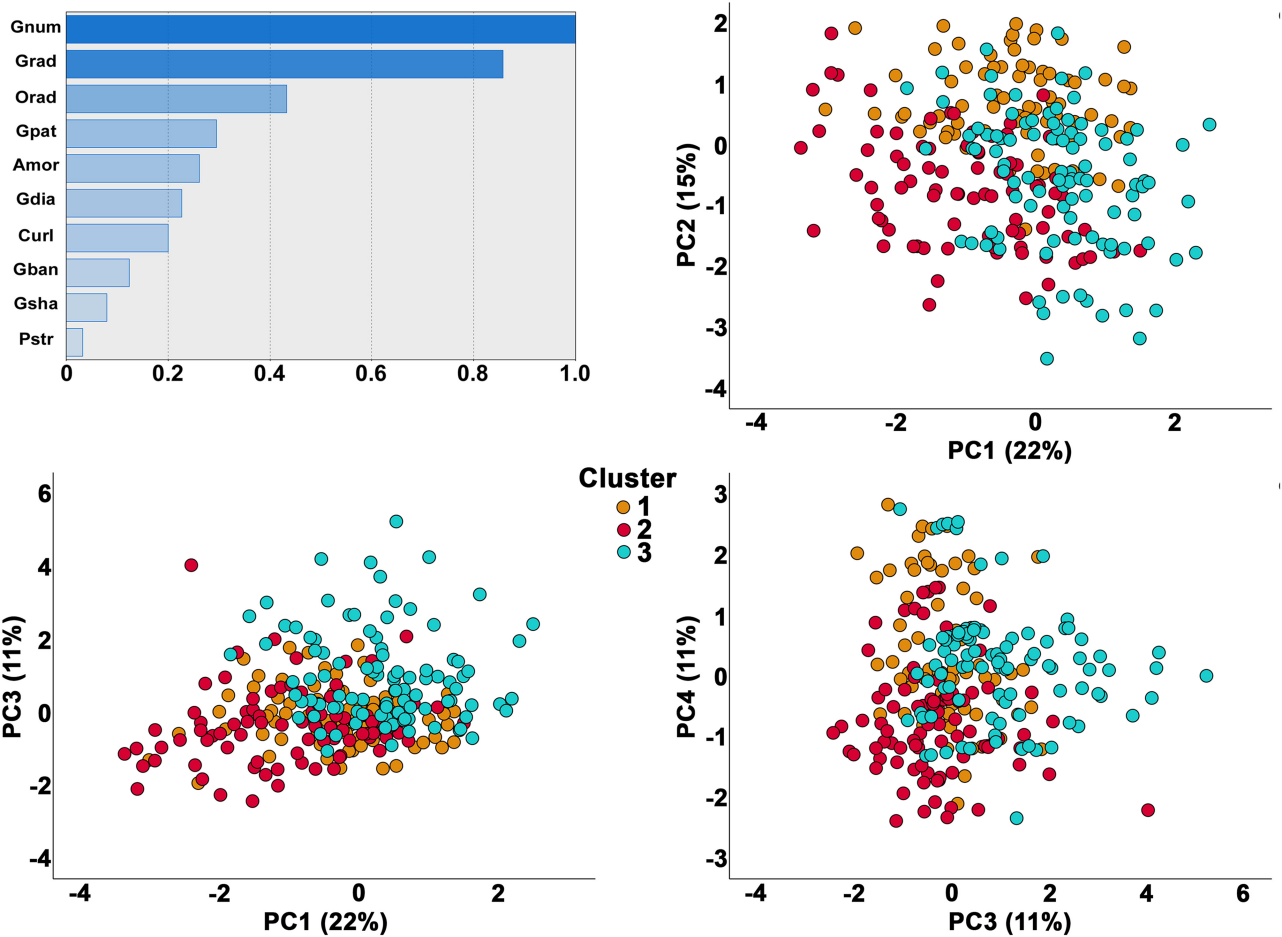
Contribution of the histological components to the clustering solution as reported from the two‐step procedure. The top‐left panel shows the index of relative importance of each histological component as identified by the two‐step cluster analysis. Representation of the three clusters in two dimensions using principal component analysis. Cluster 1 represented mostly specimens with a dominant radial organization and very numerous guttae. Cluster 2 resembled cluster 1 but with fewer guttae. Cluster 3 was dominated by specimens with a large amount of extracellular matrix forming a central plaque. Three graphs are presented showing the partial separation of the clusters, irrespective of the dimensions chosen.

Two‐step clustering revealed three distinct clusters, as depicted in Figure 4 and Table 2. The clustering effect was nevertheless weak (silhouette = 0.2), and the importance of predictive factors was variable. Cluster 1, representing radial organization (211 cases, 42%), encompassed DMs with high scores in radial guttae and other structures and a moderate score in peripheral striae which are also by definition radially organized. It was dominated by an overall organization with a confluent centre surrounded by a ring of dense guttae, then by a periphery with few guttae (sub‐type 3, Table 1). Cluster 2 (116 cases, 23%) was similar to cluster 1 but had a lower number of and smaller guttae, and less often central ECM. Cluster 3 (173 cases, 35%) had the larger amount of guttae of all sizes and was dominated by large plaques of curly and amorphous fibres covering central confluent guttae. We found no difference between clusters 1, 2, and 3 in terms of sex ratio, with 69%, 65%, and 69% of women, respectively (*χ* ^2^, *p* = 0.780), but a significant difference was noted in age, with 70 ± 8 (range 46–91), 73 ± 10 (range 42–93), and 70 ± 10 (range 41–95) years old (*p* = 0.002, Kruskal–Wallis and Dunn's *post hoc* tests), with cluster 2 being significantly older than the other two.

**Table 2:** Histological characteristics in the three clusters extracted by the two‐step clustering method.

		Cluster 1 *n* (%)	Cluster 2 *n* (%)	Cluster 3 *n* (%)	
		211 (42%)	116 (23%)	173 (35%)	
		Radial profile	Scattered guttae	ECM profile	*p* value
Gpat	Centripetal gradient	27 (12.8)	48 (41.4)	16 (9.2)	< 0.001
	Rich periphery	8 (3.8)	1 (0.9)	17 (9.8)	
	Homogeneous	0 (0)	16 (13.8)	17 (9.8)	
	Intermediate ring	175 (82.9)	48 (41.1)	117 (67.6)	
	Unclassifiable*	1 (0.5)	3 (2.6)	6 (3.5)	
Gnum	Free surface > surface covered by guttae	0 (0)	43 (37.1)	25 (14.5)	< 0.001
	Free surface = surface covered by guttae	162 (76.8)	62 (53.4)	7 (4.0)	
	Free surface < surface covered by guttae	49 (23.2)	11 (9.5)	141 (81.5)	
Gsha	Only or mostly round	196 (92.9)	106 (91.4)	131 (75.7)	< 0.001
	Balanced mixture of round and deformed guttae	15 (7.1)	9 (7.8)	37 (21.4)	
	Only or mostly deformed*	0 (0)	1 (0.9)	5 (2.9)	
Gdia	Only or mostly small	8 (3.8)	28 (24.1)	2 (1.2)	< 0.001
	Balanced mix of small and large diameters	203 (96.2)	85 (73.3)	160 (92.5)	
	Only or mostly large*	0 (0)	3 (2.6)	11 (6.4)	
Curl	Absent	31 (14.7)	52 (44.8)	17 (9.8)	< 0.001
	Present but not the dominant structure	163 (77.3)	58 (50.0)	129 (74.6)	
	Abundant	17 (8.1)	6 (5.2)	27 (15.6)	
Amor	Absent	29 (13.7)	35 (30.2)	1 (0.6)	< 0.001
	Present but not the dominant structure	181 (85.8)	79 (68.1)	149 (86.1)	
	Abundant	1 (0.5)	2 (1.7)	23 (13.3)	
Grad	Absent	2 (0.9)	17 (14.7)	5 (2.9)	< 0.001
	Present in one or two quadrants (= 90° or 180°)	12 (5.7)	89 (76.7)	33 (19.1)	
	Present in more than two quadrants (> 180°)	197 (93.4)	10 (8.6)	135 (78.0)	
Orad	Absent	0 (0)	3 (2.6)	21 (12.1)	< 0.001
	Present in one or two quadrants (= 90° or 180°)	4 (1.9)	39 (33.6)	69 (39.9	
	Present in more than two quadrants (> 180°)	207 (98.1)	74 (63.8)	83 (48.0)	
Pstr	Absent	126 (59.7)	80 (69.0)	131 (75.7)	0.009
	Present in one or two quadrants (= 90° or 180°)	54 (25.6)	27 (23.3)	31 (17.9)	
	Present in more than two quadrants (> 180°)	31 (14.7)	9 (7.8)	11 (6.4)	
Gban	Absent	163 (77.3)	111 (95.7)	164 (94.8)	< 0.001
	Present	48 (22.7)	5 (4.3)	9 (5.2)	

Amor, amorphous ECM; Curl, curly ECM; Gban, bands of guttae; Gdia, guttae diameter; Gnum, guttae number; Gpat, guttae global pattern; Grad, guttae radially aligned; Gsha, guttae shape; Orad, other structures radially aligned; Pstr, peripheral radial striae.

* Excluded from statistical analysis by *χ* ^2^ test.

### Histological phenotypes by manual classification

We isolated five main groups or dominant phenotypes: (1) round or elliptical central structure where the guttae were covered with ECM (corresponding to the conventional conception of FECD): single‐centre group for 54.0% of cases; (2) organization in radial lines (an organization that we recently described [10]): radial group for 27.6% of cases; (3) organization in several epicentres: epicentre group; (4) presence of guttae only without added ECM, and without a clearly organized central zone: the guttae‐only group, comprising 9.4% of cases. This was the most isolated group, with only seven cases in common with the radial group and one case with the fused group; and (5) fusion of guttae: fused group. This was the least independent group, with only two ‘pure’ cases and 26 cases sharing other morphological characteristics.

**Figure 5:**
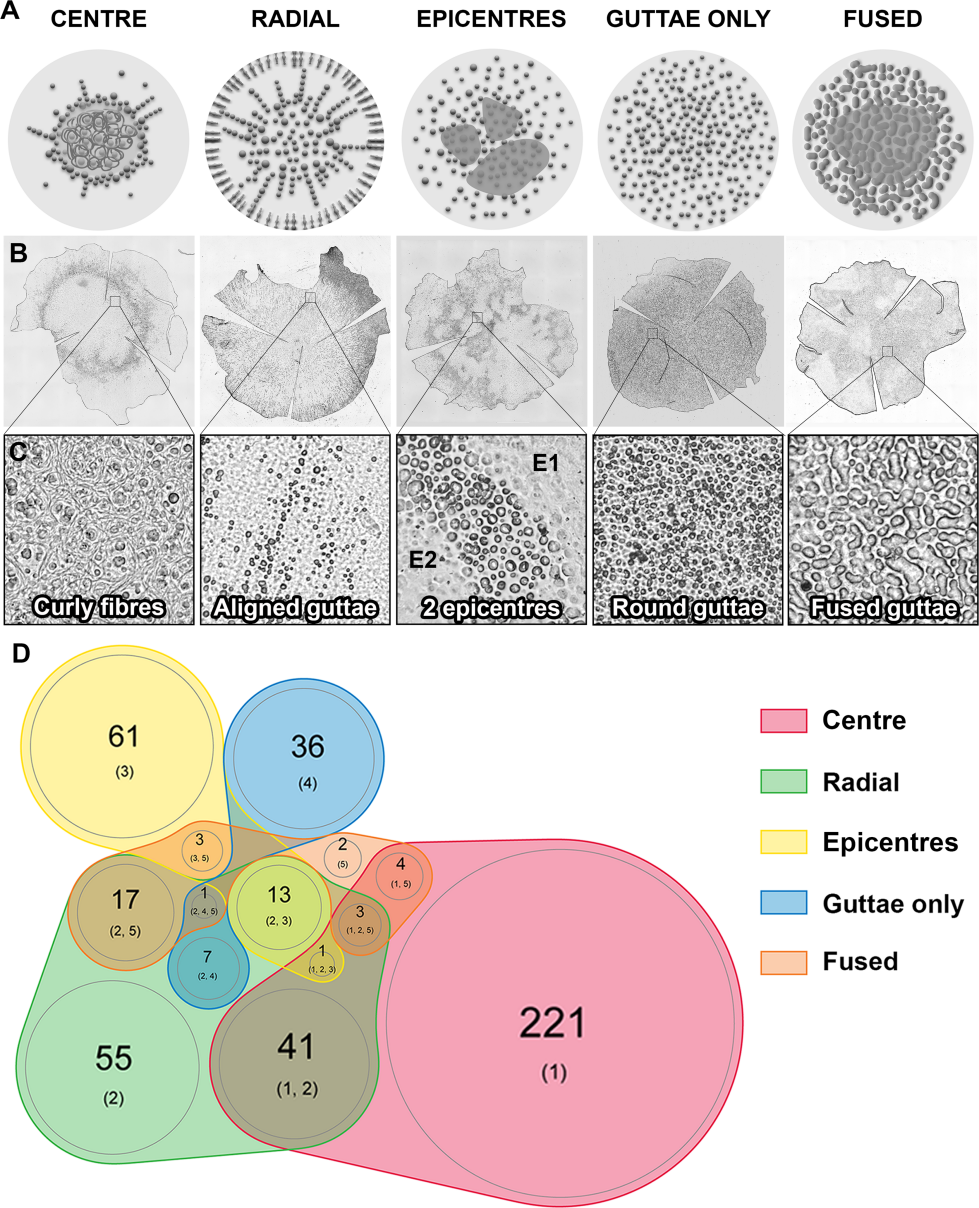
The five histological phenotypes of Fuchs endothelial corneal dystrophy identified by manual classification. (A) Schematic representation of the distribution of histological lesions in the central 8 mm of the endothelium (grey disc). (B) Descemet's membrane of FECD patients for each of the five phenotypes (low magnification ×6.4): from left to right, phenotype 1, single centre with curly fibres and guttae ring; phenotype 2, radial distribution with striae and bands of guttae; phenotype 3, epicentres with spots of curly fibres; phenotype 4, round guttae only; and phenotype 5, central fused guttae and peripheral round guttae. (C) High magnification (×40) of lesions characteristic of each histological phenotype. The images were obtained by transmitted light microscopy. (D) Proportional Venn diagram of our proposed histological classification.

Overall, 34 DMs (6.8%) could not be classified. For the remaining 466, the groups are presented in Figure 5. Eighty per cent of DMs were considered to have a single dominant feature, while 20% showed significant overlap between groups. Given this overlap, the age and sex distribution are expressed in Table 3 in two ways: for all patients in the five groups (with some patients counted two or three times) and for the five subgroups of patients with a unique characteristic. Regardless of the grouping method, no significant differences in mean age or sex ratio were observed between the five phenotypes.

**Table 3:** Age and gender distribution among the five phenotype groups.

Type of analysis	Phenotype	*n* (%)	Age, years; mean (SD)	Sex (% female)
All cases (*n* = 581)	Centre	263 (45.3)	70.7 (8.6)	68.8
	Radial	134 (23.0)	69.8 (9.3)	77.6
	Epicentre	77 (13.3)	68.2 (7.9)	61.0
	Guttae only	43 (7.4)	71.9 (10.1)	65.1
	Fused	30 (5.1)	66.8 (10.7)	83.3
	Outlier	34 (5.8)	72.0 (11.0)	61.8
	*p* value		0.010 (Kruskal–Wallis)	0.053 (*χ* ^2^)
Only cases with unique characteristics (*n* = 403)	Centre	218 (54.1)	71.9 (8.6)	65.0
	Radial	53 (13.2)	72.4 (9.0)	73.6
	Epicentre	61 (15.1)	68.6 (7.7)	58.3
	Guttae only	35 (8.7)	71.9 (10.7)	68.6
	Fused	2 (0.5)	66.0 (8.5)	50.0*
	Outlier	34 (8.4)	72.0 (11.0)	61.8
	*p* value		0.066 (Kruskal–Wallis)	0.518 (*χ* ^2^)

* Excluded from statistical analysis due to insufficient sample size.

### Relationship between phenotype and triplet repeats in the *TCF4* gene

**Figure 6:**
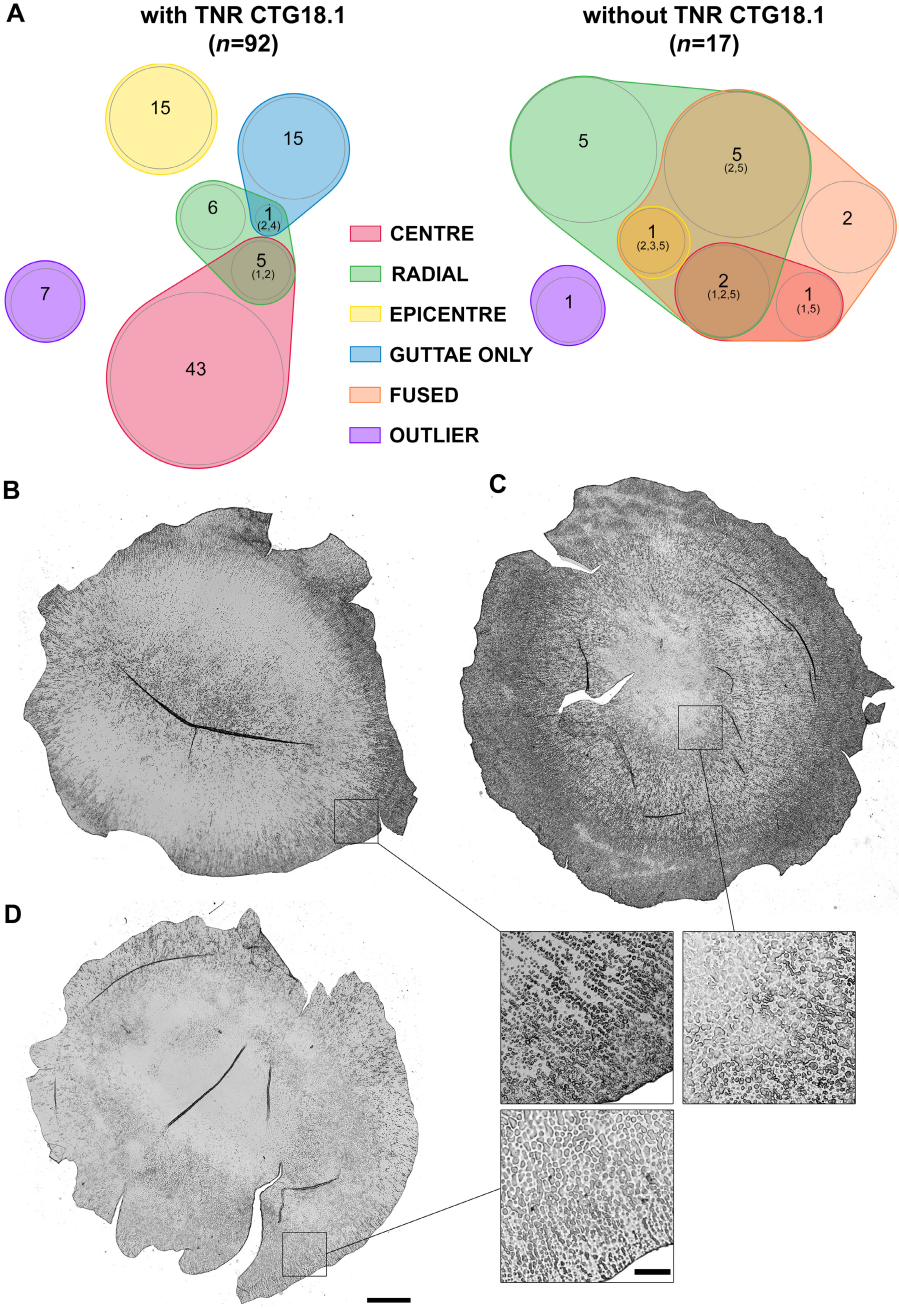
Distribution of phenotypes according to the presence or absence of CTG 18.1 trinucleotide repeat (TNR) in the *TCF4* gene. (A) The distribution was different between the two groups as shown by the Venn diagrams (*p* < 0.001). Three prototypical examples of pathological phenotypes predominant in patients without CTG18.1 TNR. (B) Eighty four‐year‐old woman presenting with radial organization and peripheral striae over 360°. (C) Seventy six‐year‐old woman presenting with a combination of radial plus fused guttae and extremely dense peripheral striae over 360°. (D) Fifty nine‐year‐old woman presenting with fused guttae and peripheral striae. Scale bar, 1 mm. Bottom right: 1 mm^2^ inset showing characteristic lesions, including fused guttae and peripheral striae. Scale bar, 200 μm.

Of the 109 FECD patients genotyped, 84.4% had TNR expansion (Table 4). The distribution of histological phenotypes according to the TNR expansion (without separating heterozygous and homozygous) is detailed in Figure 6. The phenotypes of the two groups were significantly different (*χ* ^2^ test with Yates correction, *p* < 0.001). Notably, for the 17 DMs from patients without TNR expansion, the phenotypes included only radial and/or fused components. In addition, they significantly presented peripheral striae more frequently than those with TNR expansion: 16/17 (94%) versus 45/92 (49%) (*χ* ^2^ test, *p* < 0.001). The striae were also more extensive over 360°: 10/16 (63%) versus 10/45 (22%) (*χ* ^2^ test, *p* < 0.001). Conversely, no patients with TNR expansion had fused guttae.

**Table 4:** CTG18.1 trinucleotide repeat lengths in the *TCF4* gene among FECD patients.

Number of repeats		Homozygote	Heterozygote	Total	%
Expansion‐positive (> 50)	< 100	6	27	33	30.3
	> 100	3	56	59	54.1
Intermediate	40–50	0	2	2	1.8
Expansion‐negative	< 40*	2	13	15	13.7

* Of which 11 had TNR values less than 32, meeting the RNA focus formation threshold according to Zarouchlioti *et al* [21].

## Discussion

The high prevalence of late‐onset FECD in people aged over 40 years, its increase linked to aging [22], and new short‐ and medium‐term therapeutic alternatives will require new diagnostic criteria to personalize care. The existence of a diversity of clinical forms and evolutionary profiles, observed and debated by clinicians [23], has, to our knowledge, not yet been linked to different histological forms. Based on a large series of cases, we analyse the elementary lesions characteristic of FECD and propose a new classification.

FECD is considered a disease of the central endothelium that gradually spreads towards the periphery through the accumulation of guttae and premature cell death. The historical and still most widely cited classification by Krachmer [24] defines six stages of increasing severity but does not distinguish between different clinical and/or histological forms. It is based on the number of guttae and the area of confluent guttae, and places the onset of oedema in the final stage when the confluent plaque exceeds 5 mm in diameter [2]. However, some corneas present oedema without having such a large confluent area [25]. In addition, the concept of guttae confluency remains unclear among clinicians due to the difficulties in distinguishing areas with numerous guttae in contact from areas with guttae buried/covered by the PFL. In the present work, we interpreted confluency strictly as guttae touching each other. As illustrated in a Venn diagram (Figure 5), the radial phenotype rarely presents confluent guttae (only 41/138 = 30% share characteristics with the centre group, where most cases with confluent guttae are grouped), whereas the guttae‐only phenotype never does (0/44).

Our series is representative of the conventional understanding of FECD at the surgical stage: it includes mostly women, and patients were most often aged between 60 and 70 years during operation, and the majority presented with numerous guttae, confluent in the centre. However, our systematic analysis reveals, for the first time to our knowledge, other histological forms that differ from this usual prototype.

The diameter of the guttae is the first element that allows different groups to be defined, as we had already assumed by measuring the diameter and height of three different guttae types using chromatic confocal microscopy [26]. It is generally accepted (but not formally demonstrated) that guttae diameter increases over time as severity increases [27]. The different types of guttae that we have identified suggest significant variations in their formation and growth mechanisms: While the majority (90%) of DMs contain guttae of different diameters, suggesting different durations of evolution and/or different speeds of ECM deposits, DMs from the guttae‐only group contain tens of thousands of guttae with virtually identical small diameters, consistent with the hypothesis of synchronized development and showing that severe forms of FECD exist without large guttae.

We highlight a very high frequency of ECM elements with a radial arrangement which we previously described clinically [10, 28]. Peripheral striae constitute a new structure in FECD. They have been overlooked until now probably for two reasons: (1) the endothelial periphery is difficult to visualize with a slit lamp, often obscured by the arcus senilis, and non‐contact specular microscopy is limited to the central 6 mm; and (2) they are invisible on conventional cross‐sectional histology. The peripheral striae appeared to be pathological extensions of the shorter striae that we described at the extreme periphery of healthy corneas from elderly donors [9]. Our work clearly demonstrates that FECD is not limited to the centre of the endothelium.

We assume that all these linear and radial structures could be traces left in the DM during cell migration. Normal and diseased CECs secrete ECM proteins, metalloproteases, and their endogenous tissue inhibitors, which modulate the tissue [29]. Different cells could produce different structures: normal CECs for DM structures that are not very prominent (embossments that may correspond to the location of the cell nucleus) and furrows that also exist in healthy cornea donors [9], and abnormal CECs responsible for the formation of guttae and peripheral striae along their path. In this context, two recent concepts should be considered: the existence of several subpopulations of CECs [30, 31] and a possible increase in FECD CECs’ *in vitro* migration speed, particularly in cases with TNR expansion [32, 33]. Since these alignments are found both in the centre and at the periphery and are also consistent with embryology (cells migrate from the periphery of the presumptive cornea to the centre) [34] and with the existence of endothelial progenitors and stem cells located in the extreme periphery [9, 35, 36, 37], we assume that this migration is centripetal. In addition, the frequent ‘epicentre’ phenotype could be explained by a heterogeneous distribution of abnormal peripheral CECs and/or cells which either do not migrate exactly towards the centre of the cornea or not at the same speed.

Our work provides new insight into the structure of the PFL that we identified in approximately 80% of cases. In a comparable German series of severe cases, this layer was present in 84% of 50 patients and was associated with a lower central endothelial cell density than in specimens without the layer [15]. Our work highlights that the PFL can comprise two types of abnormal ECM. It is either highly organized in the form of curly or combed fibres forming a network around and above the guttae, or amorphous, masking the buried guttae. These two forms of ECM can co‐exist. It should be noted that the term ‘curly structure’ was first used in 1974 to describe a similar organization located between Hassall–Henle bodies at the extreme periphery of healthy endothelium [18]. The existence of these two forms of ECM could correspond to different isoforms, by homology with the different deposits of transforming growth factor‐β‐induced protein mutated in epithelial‐stromal dystrophies [38]. In addition, we have just described that the PFL contains specific proteins (tenascin‐C and biglycan) [39, 40]. Further analysis will be needed to compare proteins in the two types of ECM.

We were struck by the similarities between the periphery of healthy endothelium and the centre of certain FECD groups (centre, radial, epicentre): both have excrescences in the DM (Hassall–Henle warts and guttae), striae [9], and curly fibres or structures [18, 41]. Their common feature is the presence of poorly differentiated CECs in the healthy periphery (progenitor) [9, 42] and dedifferentiated cells in the centre through endothelium–mesenchymal transition mechanisms in FECD [43].

Our two classifications do not lead to the same groupings, but they do have in common the separation of the centre and radial phenotypes. Manual classification is necessarily more diverse, since our aim was to group together cases that were broadly similar without considering the individual scores of each of the ten lesions. It allows five distinct phenotypes to be separated, even though 20% of cases share common characteristics suggesting common pathophysiological mechanisms. Interestingly, FECDs with only non‐confluent small guttae (guttae only) constitute a rare (9.4%) and virtually pure group. It is highly unlikely that this phenotype is an early stage of a ‘centre’ form, since patients were the same age and guttae covered the entire DM, whereas centre forms most often have a ring with few guttae.

Our subgroup of 109 genotyped patients includes 84.4% of patients with TNR expansion (comparable to that reported by Wieben *et al* [7] and recently by Liu *et al* [44]). To our knowledge, this is the first time that the absence of TNR expansion has been associated with a limited number of typical phenotypes: radial and/or fused guttae, absence of a central PFL (i.e. with perfect visibility of guttae) with hypertrophy of the peripheral striae. This innovative description suggests that the response of CECs to stress already identified in FECD pathogenesis varies significantly depending on their genetic background: enough to alter the formation and distribution of guttae but not enough to suppress guttae formation. The exact mechanism of deposition in the form of guttae rather than a uniform layer remains unclear.

This study has several limitations: (1) We have no clinical data and the stage of the disease at the time of transplantation may vary from one patient to another. A second prospective study is underway to analyse the clinical characteristics of our proposed histological subgroups (NCT05742321). (2) We did not study the cellular aspect for two reasons: the surgical trauma is significant and does not allow for a complete view of intact CECs, and we chose to focus first on ECM complexity. (3) Despite its relative transparency, some guttae buried in the PFL may remain invisible. (4) The PCA is not very effective, as the four PCs explain only 59% of the variance contained in the ten variables. This suggests that our interpretation was not sufficiently discriminating. This could be due to the use of three different pre‐analytical conditions (PFA, BSS, or water). We note, however, that the PCA helped us to determine that variable groupings exist and that they can separate very different forms of FECD. Automated image analysis methods should improve clustering. (5) We have not yet investigated the much rarer mutations [45], or the role of epigenetic modifications [46]. Nevertheless, given the extreme rarity of these mutations [44] on the one hand, and the relative homogeneity of the phenotypes found in the non‐TNR group on the other, an association is highly unlikely.

In summary, this large FECD series enabled us to describe new tissue structures and demonstrate the predominance of radially aligned structures, suggesting the existence of centripetal cell migration in their genesis. We propose a new histological classification into five distinct phenotypes, some of which exist only in the presence of TNR expansion (guttae only) and others that are significantly more frequent when TNRs are absent (fused guttae), suggesting different pathophysiological mechanisms.

## Author contributions statement

GTh, J‐MP, OD‐C, SP and PG conceptualized and planned the experiments. GTh secured funding. HV and GTh conducted histopathology and scoring, supervised the project and wrote, reviewed and edited the manuscript. DO and RT performed genotyping. EC, EO and GTh performed statistical analysis. HV, GTr, OD‐C, SP and ZH were responsible for tissue preparation and imaging. CM oversaw image analysis and management of the online materials. HV and FF provided critical feedback and participated in manuscript review and editing. The FFSG provided the human tissue samples. All authors reviewed the manuscript, discussed the results and contributed to its final version.

## Supporting information


**Supplementary materials** **and methods**
**Figure S1.** Surgical descemetorhexis
**Figure S2.** Representative examples of the three types of histological structures rarely described to date in Fuchs endothelial corneal dystrophy and used in our proposed classification
**Figure S3.** Genotypes for the trinucleotide repeat expansion in the *TCF4* gene
**Figure S4.** Assessment of pre‐analytical conditions on the morphology observed with transmitted light optical microscopy
**Table S1.** The French Fuchs Study Group. Twenty‐five ophthalmology departments from the following hospitals (listed in alphabetical order by hospital and including surgeons)
**Table S2.** Primers for STR‐PCR and TP‐PCR
**Table S3.** Composition of the mix used for STR‐PCR and TP‐PCR
**Table S4.** Parameters used for PCR cycles
**Table S5.** Influence of the pre‐analytic medium on the assessment of the ten Descemet's membrane characteristics

## Data Availability

The data that support the findings of this study are available from the corresponding author upon reasonable request. In addition, the collection of 500 images is publicly available in the Zenodo repository at https://doi.org/10.5281/zenodo.18335072, https://doi.org/10.5281/zenodo.18340536, https://doi.org/10.5281/zenodo.18340577, https://doi.org/10.5281/zenodo.18340671, and https://doi.org/10.5281/zenodo.18340673.

## Appendix A

The French Fuchs Study Group

Twenty‐five ophthalmology departments of the following hospitals:

University Hospital of Grenoble, France

Hôpital National des 15‐20, IHU ForeSight, GRC 32, Transplantation et Thérapies Innovantes de la Cornée, TTIC, INSERM‐DGOS CIC 1423, Paris, France

University Hospital, Nouvel Hôpital Civil, Strasbourg, France

University Hospital Cochin, Paris, France

University Hospital, Gabriel Montpied Clermont‐Ferrand, France

University Hospital Morvan, Brest, France

University Hospital of Dijon, France

University Hospital of Montpellier, France

University Hospital, Robert Debré, Reims, France

University Hospital Sart Tilman, Liège, Belgium

Kleber Ophthalmology Centre, Lyon, France

University Hospital, Purpan, Toulouse, France

University Hospital of Besançon, France

University Hospital of Saint‐Etienne, France

Clinique Monticelli‐Vélodrome Marseille, France

Ophthalmology Centre, Lausanne, Switzerland

University Hospital Charles Nicolle, Rouen, France

Ophthalmology Centre, Valence, France

Hospital Centre Metz‐Thionville, France

University Hospital Caen/Normandie, France

Hospital Centre, Antibes Juan les Pins, France

Rothschild Foundation Hospital, Paris, France

Institut ophtalmologique de l'ouest, clinique Jules Verne, Nantes, France

University Hospital Pellegrin, Bordeaux

University Hospital, Hôtel Dieu, Nantes, France

Dr Diane Bernheim; Prof Christophe Chiquet; Prof Vincent Borderie; Prof Tristan Bourcier; Prof Jean‐Louis Bourges; Prof Frédéric Chiambaretta; Prof Béatrice Cochener; Prof Louis Arnould; Dr Florian Baudin; Prof Catherine Creuzot; Prof Vincent Daien; Prof Alexandre Denoyer; Prof Bernard Duchesne; Dr Nicolas Duquesne; Prof Pierre Fournie; Prof Anne‐Sophie Gauthier; Prof Philippe Gain; Prof Gilles Thuret; Prof Louis Hoffart; Dr François Majo; Prof Marc Muraine; Dr Romain Mouchel; Dr Jean Marc Perone; Prof Jean Claude Quintyn; Dr Alexandra Rabot; Dr Alain Saad; Dr Damien Gatinel; Dr Pierre‐Yves Santiago; Dr Jean‐Michel Bosc; Prof David Touboul; Dr Bertrand Vabres. (see supplementary material, Table S1).
