# Supplementary material for: Pathological classification of Fuchs endothelial corneal dystrophy into several types and their relationships with CTG18.1 expansion repeats
Source: J Pathol. 2026 Feb 25;269(2):182–96. doi: 10.1002/path.70044 (PMC13140138; doi:10.1002/path.70044)
Supplement: Supplementary file 1 — Supplementary materials and methods Figure S1. Surgical descemetorhexis Figure S2. Representative examples of the three types of histological structures rarely described to date in Fuchs endothelial corneal dystrophy and used in our proposed classification Figure S3. Genotypes for the trinucleotide repeat expansion in the TCF4 gene Figure S4. Assessment of pre‐analytical conditions on the morphology observed with transmitted light optical microscopy Table S1. The French Fuchs Study Group. Twenty‐five ophthalmology departments from the following hospitals (listed in alphabetical order by hospital and including surgeons) Table S2. Primers for STR‐PCR and TP‐PCR Table S3. Composition of the mix used for STR‐PCR and TP‐PCR Table S4. Parameters used for PCR cycles Table S5. Influence of the pre‐analytic medium on the assessment of the ten Descemet's membrane characteristics [file PATH-269-182-s001.docx]

**Pathological classification of Fuchs endothelial corneal dystrophy into several types and their relationships with CTG18.1 expansion repeats**

H Vaitinadapoulé *et al. J Pathol* [https://doi.org/10.1002/path**.**70044](https://doi.org/10.1002/path.70044)

**Supplementary materials and methods**

**Supplementary Figures S1–S4**

**Supplementary Tables S1–S5**

Reference numbers refer to the main text list

**Supplementary materials and methods**

*Ethics approval*

The handling of tissues adhered to the tenets of the Declaration of Helsinki (2024 revision) [17], ensuring donor confidentiality, and was approved by the Ethics Committee of the St-Etienne University Hospital (IRB_IORG0007394, Ref_IRBN1142021/CHUSTE).

*Human tissue samples*

Human tissue samples were collected from 25 hospitals by 29 surgeons (supplementary material, Table S1). In this study of surgical specimens, the only clinical data authorized for collection were age and sex.

*Methodological details for genotyping*

Blood samples were collected at the hospitals of Saint-Étienne and Metz. CTG18.1 TNR expansion in the *TCF4* gene was quantified using short tandem repeat-polymerase chain reaction (STR-PCR) and triplet-primed polymerase chain reaction (TP-PCR) as previously described [45]. Genetic analyses were conducted at the Molecular Genetics Laboratory of the Saint-Étienne University Hospital (CHU). Based on the data from Wieben *et al*, the genetic profiles were classified into three groups: expansion-positive (> 50 repeats), intermediate (40–50 repeats), and expansion-negative (< 40 repeats) [7]. STR-PCR is capable of precisely detecting up to 70–80 CTG18.1 TNR expansions, while TP-PCR enables the detection of large-sized repeated alleles. However, beyond 100 repeats, it can only confirm the presence of a large allele without determining the exact number of repeats. The primers, PCR mixes, and cycle conditions were as previously described by Mootha *et al* [47] and are listed in supplementary material, Tables S2–S4.

STR-PCR involves amplifying a specific DNA sequence that contains a short nucleotide sequence repeated several times using two primers flanking the repeat: P1 labelled with a fluorochrome (5' FAM) and P2 (supplementary material, Table S2). Capillary electrophoresis is then used to separate the fluorescent PCR products containing the repeat, allowing identification of the repeat size in each allele. TP-PCR also uses two primers flanking the repeat: P1 labelled with a fluorochrome (5' FAM) and P3, along with an additional internal primer, P4, specific to the CTG18.1 repeat. The P4 primer has a 5' tail sequence corresponding to primer P3. This 5' tail of P4 has no homology with the human sequence (supplementary material, Table S2). P4 binds to multiple sites within the CTG repeat during the initial amplification cycles, limiting the formation of secondary structures. Primer P3, which hybridizes to fragments containing primer P4, selectively continues the amplification of these obtained fragments. Capillary electrophoresis is then used to separate the fluorescent PCR products containing the repeat, allowing the identification of the repeat size in each allele. Genomic DNA was extracted from peripheral blood leukocytes followed by a quality check. It was then diluted to a final solution with a concentration of 50 ng/µl. The primers used for STR-PCR and TP-PCR were as described by Mootha *et al* and are shown in supplementary material, Table S3 [47]. PCR mixes were prepared with the QIAGEN HotStarTaq DNA Polymerase kit (QIAGEN S.A.S., Courtaboeuf, France) with the concentrations used for each mix listed in supplementary material, Table S3. The PCR cycles were performed using the parameters described by Mootha *et al* [47], also shown in supplementary material, Table S4.

PCR products from both techniques were analysed by capillary electrophoresis on a SeqStudio (Applied Biosystems, Thermo Fisher Scientific, Waltham, MA, USA) to separate the fluorescent fragments containing the repeat. For this, 0.5 µl/l of PCR product from one of the two techniques was mixed with 0.3 µl/l of GeneScan 500 Liz size marker and 14.2 µl/l of Hi-Di formamide. An additional denaturation step of 3 min at 86 °C was performed for the TP-PCR immediately before capillary electrophoresis migration. The results of the capillary electrophoresis were visualized using GeneMapper™ Software 6 (Thermo Fisher Scientific; https://www.thermofisher.com/order/catalog/product/4475073) to determine the size of the repeats (supplementary material, Figure S3).

*Influence of pre-analytical conditions*

The absence of morphological degradation of the Descemet’s membrane (DM) during storage was verified by comparing samples analysed on the same day with samples stored for 1–52 weeks (supplementary material, Figure S4). In water (43% of cases) or balanced salt solution (BSS) (24% of cases), corneal endothelial cells (CECs) disappeared but ECM morphology observed under optical microscopy remained intact. In paraformaldehyde (PFA) (33% of cases), residual cells persisted but their morphology was often altered compared with healthy CECs due to both the pathological process and surgical trauma (stripping and passage through a narrow incision). This fixation method was quickly abandoned as our objective was to analyse the ECM only. All ten elementary lesions and the five groups of the manual classification were observed under all three transport conditions. None of the lesions or groups disappeared under any of the three conditions. The distribution of the various lesions under the three environmental conditions is detailed in supplementary material, Table S5. For three of the ten items (amorphous ECM; guttae radially aligned; other structures radially aligned), the DMs in PFA had lower scores, suggesting that the fixed residual cells interfered with the observation of the finest structures (masking effect). For peripheral striae, samples transported in BSS showed more striae, but these had a larger Feret diameter (supplementary material, Table S5), reflecting a centre effect as some ophthalmology centres that produced larger descemetorhexis were inadvertently supplied only with collection tubes pre-filled with BSS.

Analysing the samples transported under these three conditions helped to avoid bias (e.g. potential tissue shrinkage in a fixative or potential artefactual degradation in the absence of fixation) but could have made reading more difficult (addition of noise) particularly due to fixed and unevenly distributed residual cells, which could cause certain details to be missed.

**Supplementary Figures**

**Human tissue samples:** Descemetorhexis


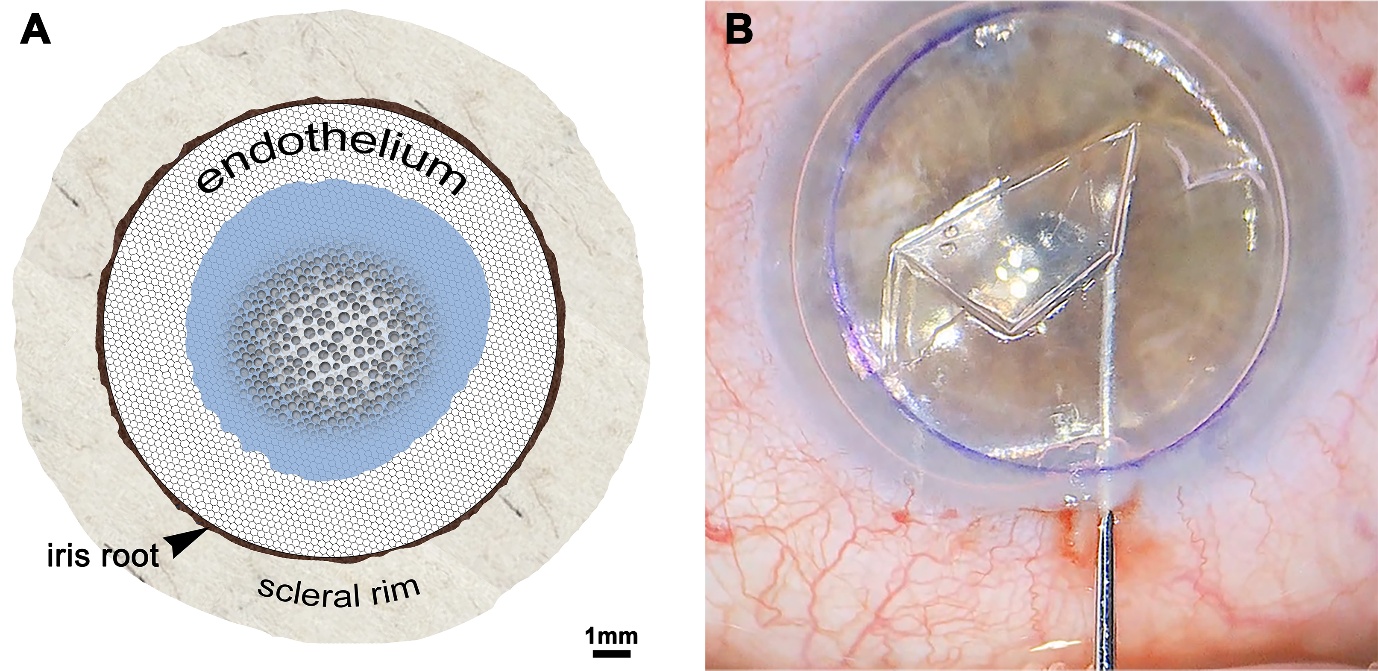


**Figure S1. Surgical descemetorhexis.** (A) Schematic diagram of human corneo-scleral tissue (as for instance is obtained from a donor cornea) seen by the inner side. The approximately 13 mm diameter endothelium (covered by hexagonal cells) is much larger than the tissue removed during surgery (descemetorhexis, in blue) and analysed in this work. Most of the guttae are in the centre of the tissue. (B) Intra-operative view of the 8 mm diameter descemetorhexis step. The surface violet mark (8 mm) helps the surgeon to tear the Descemet membrane at the desired size. The diameter of the endothelium is larger than it seems from a surface view because it extends under the non-transparent sclero-corneal limbus.


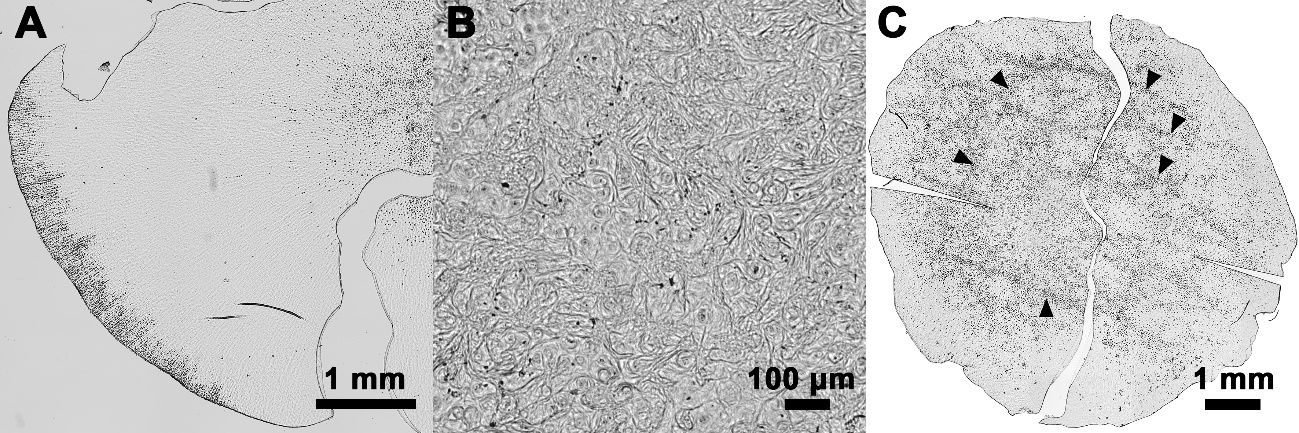


**Figure S2.** **Representative examples of the three types of histological structures rarely described to date in Fuchs endothelial corneal dystrophy and used in our proposed classification.** (A) Peripheral striae composed of guttae-like excrescences disposed radially and, by definition, interrupted by the border of the specimen, indicating that they extended more peripherally. (B) Curly fibres corresponding to a well-organized extracellular matrix located in the posterior fibrillar layer. (C) Guttae bands (arrowheads) formed by guttae that are disposed more densely than everywhere along few long lines.

**
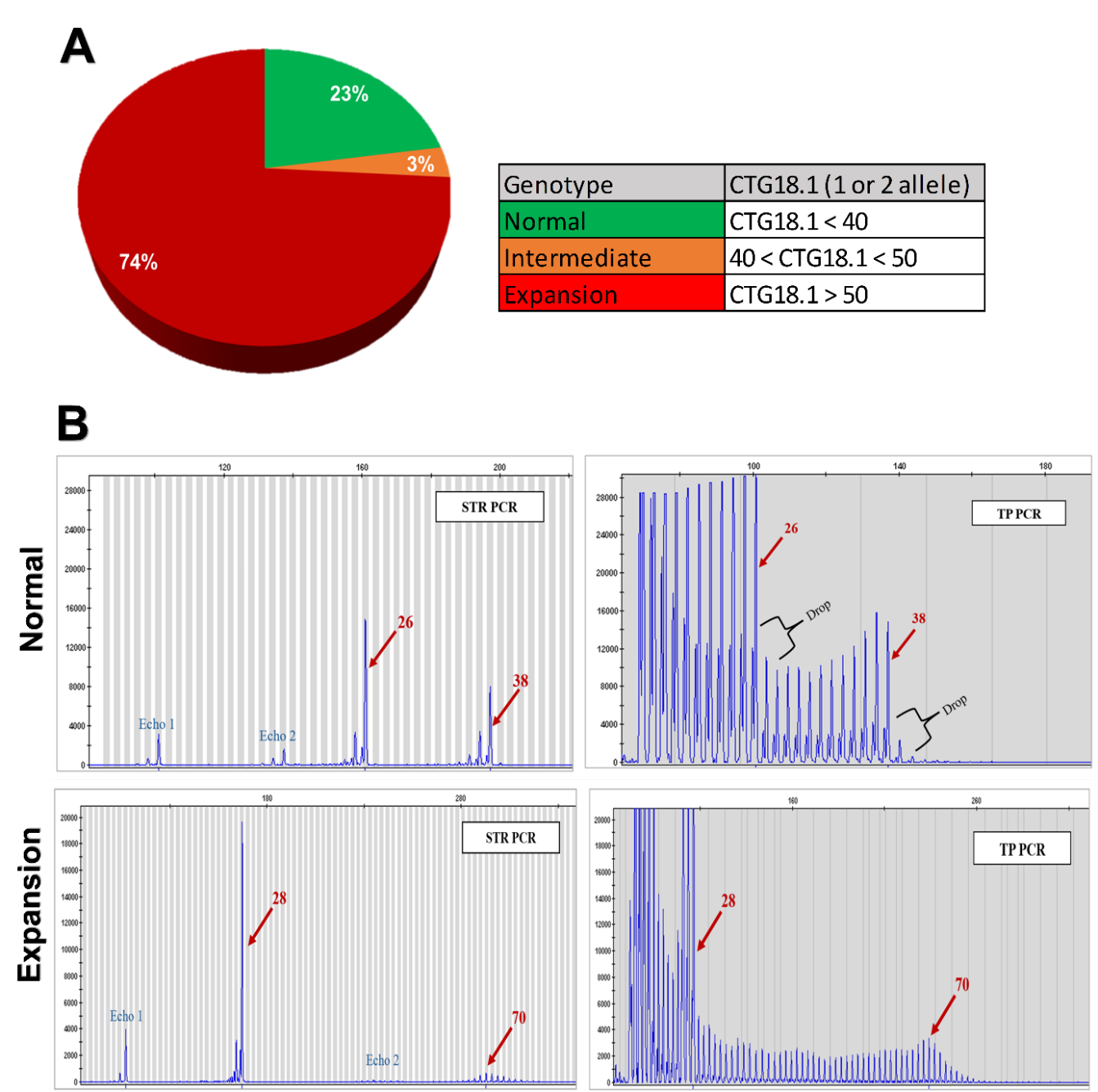
**

**Figure S3. Genotypes for the trinucleotide repeat expansion in the *TCF4* gene.** (A) Genotyping of CTG18.1 trinucleoide repeat (TNR) expansion: distribution of CTG18.1 expansion among genotyped patients. Alleles with fewer than 40 repeats were classified as normal alleles; those with 40–50 repeats as intermediate alleles; and those with more than 50 repeats as alleles with CTG18.1 expansion (mutated). (B) Results of CTG18.1 expansion analysis by STR-PCR and TP-PCR for representative genetic profiles. Normal heterozygote profile (upper panels): both alleles (26 and 38 repetitions) are visible using the two techniques. A systematic ‘echo’ located 20 repeats upstream is observed for each allele in STR-PCR. In TP-PCR, the normal allele corresponds to the last peak before the ‘drop-off’. Mutated heterozygote profile (lower panels): the normal allele (28 repeats) is visible using the two techniques, but the second allele shows a broadened peak in STR-PCR. In TP-PCR, an expanded profile is detected by a characteristic train of peaks, confirming the pathological expansion and allowing repeat sizing for the second allele (70 repeats).


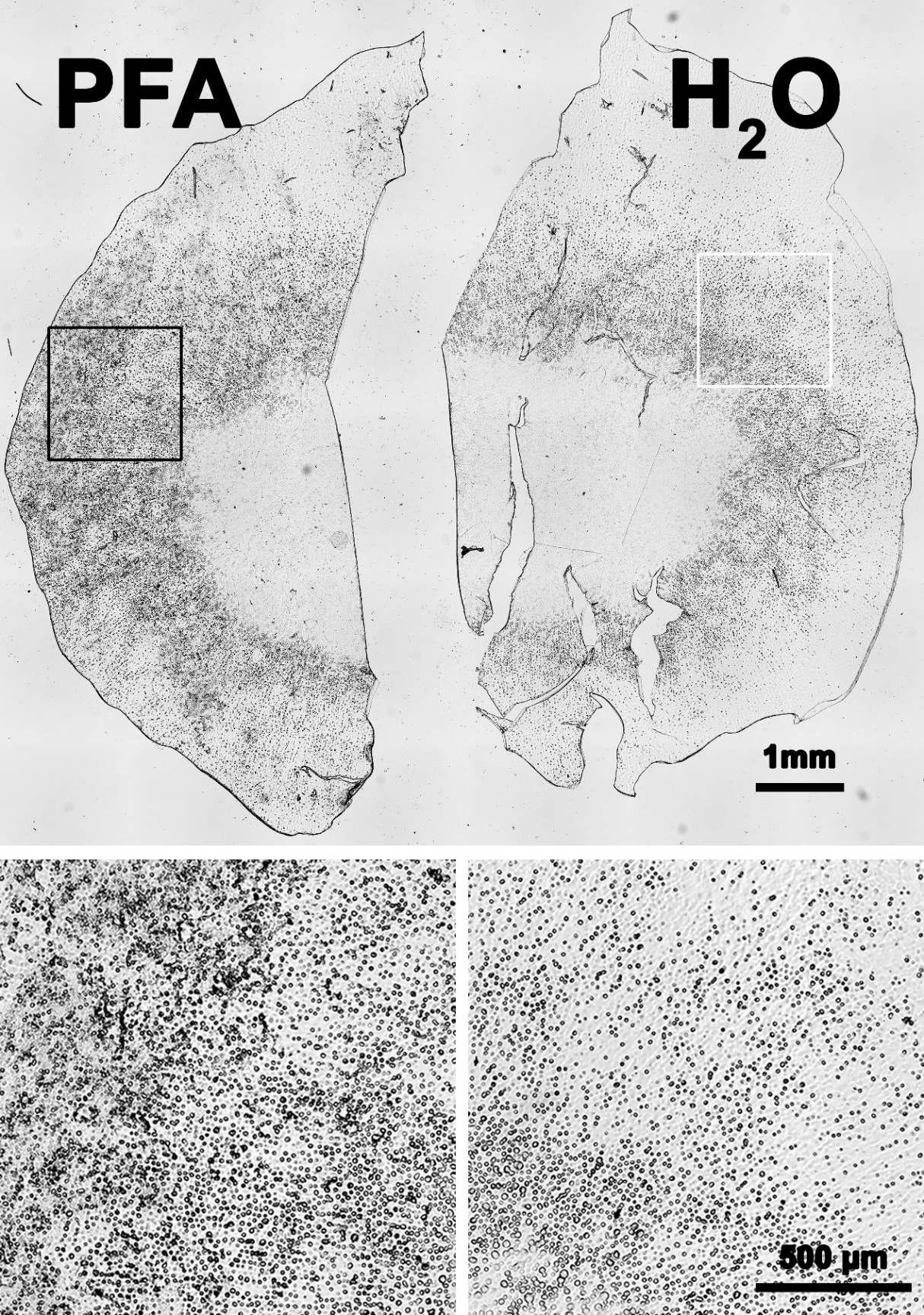


**Figure S4.** **Assessment of pre-analytical conditions on the morphology observed with transmitted light optical microscopy.** The specimen fixed in paraformaldehyde (PFA) contained areas of preserved endothelial cells forming greyish veils covering guttae at different locations as well as cell debris forming black spots or lines masking several guttae. Most of the surface was nevertheless almost decellularized, leaving access to most of the guttae. On the contrary, the specimen immersed in water appeared totally decellularized, with all guttae clearly accessible.

**Supplementary Tables**

**Table S1.** The French Fuchs Study Group. Twenty-five ophthalmology departments from the following hospitals (listed in alphabetical order by hospital and including surgeons).

| **Surgeons** | **Hospital, city** | **Country** |
| --- | --- | --- |
| Dr Diane Bernheim  Prof. Christophe Chiquet | University Hospital of Grenoble, Grenoble | France |
| Prof. Vincent Borderie | Hôpital National des 15-20, IHU ForeSight, GRC 32, Transplantation et Thérapies Innovantes de la Cornée, TTIC, INSERM-DGOS CIC 1423, Paris | France |
| Prof. Tristan Bourcier | University Hospital, Nouvel Hôpital Civil, Strasbourg | France |
| Prof. Jean-Louis Bourges | University Hospital Cochin, Paris | France |
| Prof. Frédéric Chiambaretta | University Hospital Gabriel Montpied, Clermont-Ferrand | France |
| Prof. Béatrice Cochener | University Hospital Morvan, Brest | France |
| Prof. Louis Arnould  Dr Florian Baudin  Prof. Catherine Creuzot | University Hospital of Dijon | France |
| Prof. Vincent Daien | University Hospital of Montpellier | France |
| Prof. Alexandre Denoyer | University Hospital Robert Debré, Reims | France |
| Prof. Bernard Duchesne | University Hospital Sart Tilman, Liège | Belgium |
| Dr Nicolas Duquesne | Kleber Ophthalmology Centre, Lyon | France |
| Prof. Pierre Fournie | University Hospital Purpan, Toulouse | France |
| Prof. Anne-Sophie Gauthier | University Hospital of Besançon | France |
| Prof. Philippe Gain  Prof. Gilles Thuret | University Hospital of Saint-Etienne | France |
| Prof. Louis Hoffart | Clinique Monticelli-Vélodrome, Marseille | France |
| Dr François Majo | Ophthalmology Centre, Lausanne | Switzerland |
| Prof. Marc Muraine | University Hospital Charles Nicolle, Rouen | France |
| Dr Romain Mouchel | Kleber Ophthalmology Centre, Lyon | France |
| Dr Jean Marc Perone | Hospital Center Metz-Thionville | France |
| Prof. Jean Claude Quintyn | University Hospital Caen/Normandie | France |
| Dr Alexandra Rabot | Hospital Center, Antibes Juan les Pins | France |
| Dr Alain Saad  Dr Damien Gatinel | Rothschild Foundation Hospital, Paris | France |
| Dr Pierre-Yves Santiago  Dr Jean-Michel Bosc | Institut ophtalmologique de l’ouest, Clinique Jules Verne, Nantes | France |
| Prof. David Toubould | University Hospital Pellegrin, Bordeaux | France |
| Dr Bertrand Vabres | University Hospital Hôtel Dieu, Nantes | France |

**Table S2.** Primers for STR-PCR and TP-PCR.

| **Primer** | **Sequence 5'–3'** | **Genomic position (GRCh38)** | **Technique** |
| --- | --- | --- | --- |
| P1  (5' FAM) | AATCCAAACCGCCTTCCAAGT | chr18:55.586.242-55.586.262 | STR-PCR  TP-PCR |
| P2 | CAAAACTTCCGAAAGCCATTTCT | chr18:55.586.076-55.586.098 | STR-PCR |
| P3 | TACGCATCCCAGTTTGAGACG | No homology | TP-PCR |
| P4 | TACGCATCCCAGTTTGAGACGCAGCAGCAGCAGCAG | 5' tail – no homology  chr18:55.586.156-55.586.227 | TP-PCR |

**Table S3.** Composition of the mix used for STR-PCR and TP-PCR.

| **Reagent** | **STR-PCR**  **(µl per reaction)** | **TP-PCR**  **(µl per reaction)** |
| --- | --- | --- |
| Water | 10.5 | 7.6 |
| Buffer Q | 2.5 | 2.5 |
| dNTP (0.2 mm) | 2.5 | 2.5 |
| Solution Q | 5 | 5 |
| MgCl_2_ 1.5 mm | 1.5 | – |
| Taq Q polymerase | 0.2 | 0.2 |
| Primer P1 (5' FAM) – F | 0.4 (0.3 m solution) | 1.2 (1 m solution) |
| Primer P2 – R (0.3 m) | 0.4 | – |
| Primer P3 – R (1 m) | – | 1.2 |
| Primer P4 – R (0.03 m) | – | 0.8 |
| **DNA (50 ng/µl)** | **2** | **4** |
| **Total volume** | **25** | **25** |

**Table S4.** Parameters used for PCR cycles.

|  | **Denaturation** | **Annealing** | | | | | | | **Extension** |
| --- | --- | --- | --- | --- | --- | --- | --- | --- | --- |
| STR-PCR | 95 °C 10 min | 94 °C 30 s | | | 58 °C 30 s | | | 72 °C 30 s | 72 °C 10 min |
|  |  | 30 cycles | | | | | | |  |
| TP-PCR | 95 °C 9 min | 95 °C 30 s | 62 °C 30 s | 72 °C 4 min | | 95 °C 45 s | 62 °C 45 s | 72 °C 4 min  + 15 s extension at each cycle | 72 °C 10 min |
|  |  | 10 cycles | | | | 30 cycles | | |  |

**Table S5.** Influence of the pre-analytical medium on the assessment of the ten Descemet’s membrane characteristics. Results are expressed as *n* (%).

| **Criterion (abbreviation)** | **Grades** | **BSS** | **Water** | **PFA** | ***p* value (***χ***^2^)** |
| --- | --- | --- | --- | --- | --- |
| Guttae number  (Gnum) | 0: free surface > surface covered by guttae | 28 (23.5%) | 27 (12.7%) | 13 (7.8%) | 0.002 |
|  | 1: free surface = surface covered by guttae | 56 (46.7%) | 96 (45.1%) | 79 (47.3%) |  |
|  | 2: free surface < surface covered by guttae | 36 (30.0%) | 90 (42.3%) | 75 (44.9%) |  |
| Guttae shape  (Gsha) | 0: only or mostly round | 102 (85.0%) | 191 (89.7%) | 140 (83.8%) | N/A |
|  | 1: balanced mixture of round and deformed guttae | 18 (15.0%) | 21 (9.9%) | 22 (13.2%) |  |
|  | 2: only or mostly deformed | 0 (0.0%) | 1 (0.5%) | 5 (3.0%) |  |
| Guttae diameter  (Gdia) | 0: only or mostly small | 13 (10.8%) | 17 (8.0%) | 8 (4.8%) | N/A |
|  | 1: balanced mix of small and large diameters | 105 (87.5%) | 195 (91.5%) | 148 (88.6%) |  |
|  | 2: only or mostly large | 2 (1.7%) | 1 (0.5%) | 11 (6.6%) |  |
| Guttae overall pattern  (Gpat) | 0 centripetal gradient | 24 (20.0%) | 38 (17.8%) | 29 (17.4%) | N/A |
|  | 1 rich periphery | 8 (6.7%) | 8 (3.8%) | 10 (6.0%) |  |
|  | 2 homogeneous | 4 (3.3%) | 8 (3.8%) | 21 (12.6%) |  |
|  | 3 intermediate ring | 80 (66.7%) | 156 (73.2%) | 104 (62.3%) |  |
|  | 4 unclassifiable | 4 (3.3%) | 3 (1.4%) | 3 (1.8%) |  |
| Guttae radial orientation  (Grad) | 0: absent | 4 (3.4%) | 6 (2.8%) | 14 (8.4%) | <0.001 |
|  | 1: present in one or two quadrants (= 90° or 180°) | 36 (30.3%) | 39 (18.3%) | 59 (35.3%) |  |
|  | 2: present in more than two quadrants (>180°) | 79 (66.4%) | 168 (78.9%) | 94 (56.3%) |  |
| Guttae band  (Gban) | 0: absent | 107 (90.0%) | 178 (83.6%) | 152 (91.0%) | 0.06 |
|  | 1: present | 12 (10.0%) | 35 (16.4%) | 15 (9.0%) |  |
| ‘Curly fibers’  (Curl) | 0: absent | 27 (22.5%) | 32 (15.0%) | 41 (24.6%) | 0.153 |
|  | 1: present but not the dominant structure | 79 (65.8%) | 159 (74.6%) | 112 (67.1%) |  |
|  | 2: abundant | 14 (11.7%) | 22 (10.3%) | 14 (8.4%) |  |
| Amorphous extracellular matrix  (Amor) | 0: absent | 20 (16.7%) | 34 (16.0%) | 11 (6.6%) | 0.038 |
|  | 1: present but not the dominant structure | 91 (76.7%) | 170 (79.8%) | 147 (88.0%) |  |
|  | 2: abundant | 8 (6.7%) | 9 (4.2%) | 9 (5.4%) |  |
| Radial orientation of other structures (excluding guttae)  (Orad) | 0: absent | 3 (2.5%) | 8 (3.8%) | 13 (7.8%) | <0.001 |
|  | 1: present in one or two quadrants (= 90° or 180°) | 19 (15.8%) | 34 (16.0%) | 59 (35.3%) |  |
|  | 2: present in more than two quadrants (>180°) | 98 (81.7%) | 171 (80.3%) | 95 (56.9%) |  |
| Peripheral striae  (Pstr) | 0: absent | 54 (45.8%) | 154 (72.3%) | 128 (76.6%) | <0.001 |
|  | 1: present in one or two quadrants (= 90° or 180°) | 42 (35.0%) | 41 (19.2%) | 29 (17.4%) |  |
|  | 2: present in more than two quadrants (>180°) | 23 (19.2%) | 18 (8.5%) | 10 (6.0%) |  |
| Feret min (mean ± SD) | | 9.2 ± 0.6 | 8.0 ± 1.1 | 7.9 ± 0.9 | <0.001 |
| Feret max (mean ± SD) | | 10.5 ± 0.6 | 9.4 ± 1.1 | 9.1 ± 0.9 | <0.001 |

N/A, not applicable.
